# Optimal Scanning Protocols for Dual-Energy CT Angiography in Peripheral Arterial Stents: An *in Vitro* Phantom Study

**DOI:** 10.3390/ijms160511531

**Published:** 2015-05-20

**Authors:** Abdulrahman Almutairi, Zhonghua Sun, Zakariya Al Safran, Abduljaleel Poovathumkadavi, Suha Albader, Husam Ifdailat

**Affiliations:** 1 Department of Medical Radiation Sciences, Curtin University, Perth, 6102 Western Australia, Australia; E-Mail: aa1ss@hotmail.com; 2 Department of Medical Imaging, King Fahad Specialist Hospital, Dammam 31444, Saudi Arabia; E-Mails: doctorsafranzak@yahoo.com (Z.A.S.); drjaleelpoovathum@gmail.com (A.P.); s_albadr@yahoo.com (S.A.); hfdielat@gmail.com (H.I.)

**Keywords:** dual-energy CT, gemstone spectral imaging, image noise, monochromatic image, peripheral arterial stent

## Abstract

Objective: To identify the optimal dual-energy computed tomography (DECT) scanning protocol for peripheral arterial stents while achieving a low radiation dose, while still maintaining diagnostic image quality, as determined by an *in vitro* phantom study. Methods: Dual-energy scans in monochromatic spectral imaging mode were performed on a peripheral arterial phantom with use of three gemstone spectral imaging (GSI) protocols, three pitch values, and four kiloelectron volts (keV) ranges. A total of 15 stents of different sizes, materials, and designs were deployed in the phantom. Image noise, the signal-to-noise ratio (SNR), different levels of adaptive statistical iterative reconstruction (ASIR), and the four levels of monochromatic energy for DECT imaging of peripheral arterial stents were measured and compared to determine the optimal protocols. Results: A total of 36 scans with 180 datasets were reconstructed from a combination of different protocols. There was a significant reduction of image noise with a higher SNR from monochromatic energy images between 65 and 70 keV in all investigated preset GSI protocols (*p* < 0.05). In addition, significant effects were found from the main effect analysis for these factors: GSI, pitch, and keV (*p* = 0.001). In contrast, there was significant interaction on the unstented area between GSI and ASIR (*p* = 0.015) and a very high significant difference between keV and ASIR (*p* < 0.001). A radiation dose reduction of 50% was achieved. Conclusions: The optimal scanning protocol and energy level in the phantom study were GSI-48, pitch value 0.984, and 65 keV, which resulted in lower image noise and a lower radiation dose, but with acceptable diagnostic images.

## 1. Introduction

The increasing prevalence of peripheral artery disease (PAD) is an important cardiovascular disease risk factor [1,2,3]. Stent placement for occlusive vascular disease is recognized as a safe and effective alternative treatment for PAD [4]. The main concern of stent implantation is the development of in-stent restenosis. Recent studies have shown a 30%–55% restenosis rate after the first year of stent implementation [5,6,7], indicating that a follow-up examination for the patency of implanted stents is important. A number of imaging techniques have been used to evaluate stent patency, including digital subtraction angiography (DSA), multi-detector computed tomography (MDCT), Doppler ultrasound, and magnetic resonance imaging. Doppler ultrasound is a non-invasive technique, which allows measurement of blood flow to confirm the diagnosis of occlusive PAD. However, the use of Doppler ultrasound is restricted when vascular are calcified or stented. Its diagnostic accuracy also depends on both the operator’s experience and the patient’s body habitus. Magnetic resonance angiography, on the other hand, could represent an alternative, non-invasive approach. However, for stents evaluation this procedure might be limited due to signal decrease or signal loss caused by metallic stents. Although DSA was the standard follow-up procedure for PAD, there are some disadvantages of this modality, which include invasiveness and limited assessment to the vessel structures. Therefore, it has gradually been replaced by less invasive techniques, such as MDCT [8,9], because MDCT is associated with few procedure-related complications, but with shorter procedural time, and fewer motion artifacts [8,9]. Despite these advantages, MDCT has its weaknesses, including a higher rate of contrast medium-induced nephrotoxicity, suffering from blooming artifacts caused by stent struts, and risk of high radiation dose.

The latest MDCT systems, such as dual-source computed tomography (DSCT) and dual-energy computed tomography (DECT), are capable of addressing these weaknesses [10]. In particular, DECT has the ability to distinguish different materials at high density—for example, separating iodinated contrast from other materials [11]. In addition, beam-hardening artifacts, which usually result from the polychromatic energy of the X-ray spectrum, can be eliminated by using the monochromatic energy images (MEI) spectrum. Furthermore, shorter rotation time and use of iterative reconstruction can reduce the radiation dose [12]. Although extensive studies have been conducted on the use of DECT for cardiovascular disease, there is a paucity of literature focusing on the lower extremities, especially for stent patency evaluation [13,14,15,16].

Most of the previous studies on DECT were performed on dual-source DECT, which uses two X-ray tubes [8,16,17,18,19]. However, the fast kilovoltage switching CT scanner, using one X-ray tube and a full field of view (FOV) with special detector gemstone spectral image (GSI) of GE medical systems, represents another advantage of DECT, as it can improve image quality by reducing beam-hardening artifacts associated with stents. Furthermore, it can distinguish between materials such as contrast and soft tissue or other materials by suppressing one material and enhancing the other with better temporal registration [20]. To the best of our knowledge, there is no report available in the literature on using the fast kilovoltage switching GE scanner for evaluation of periperal arterial stents. Thus, the purpose of this study is to identify an optimal DECT scanning protocol that provides a lower radiation dose and maintains image quality in peripheral arterial stents based on an *in vitro* phantom study.

## 2. Results

**Figure 1:**
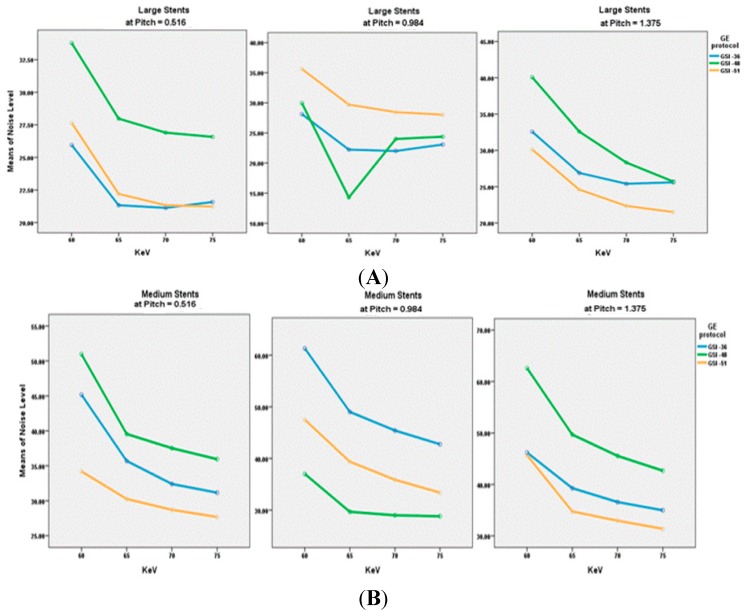

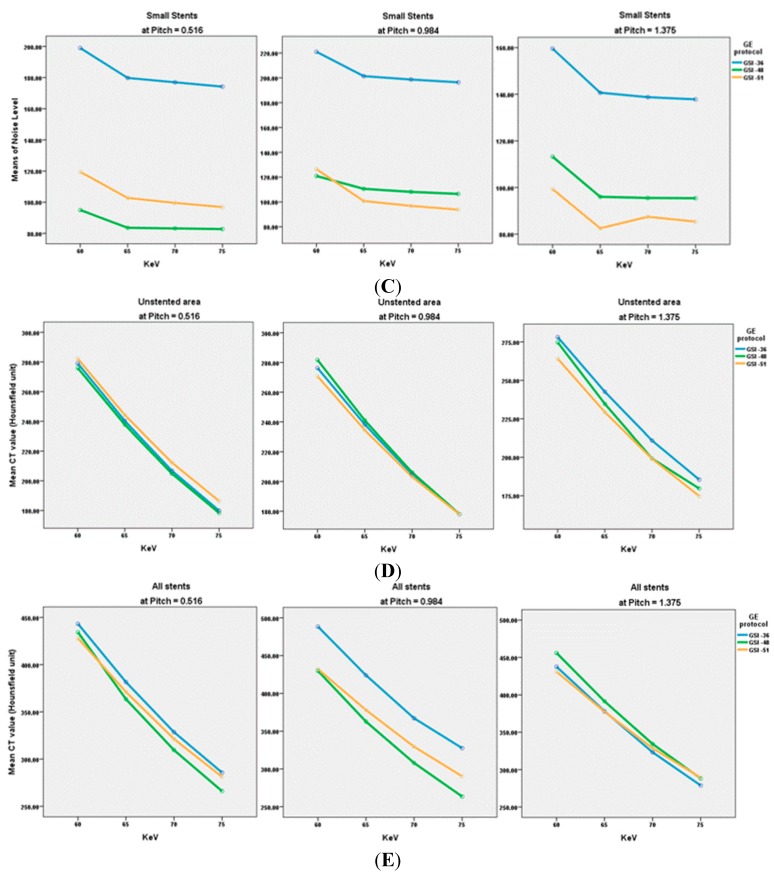
(**A**–**C**) show the comparison of Noise Level at different kiloelectron voltage (keV) with the three preset GSI protocols and three pitch values, while Figure (**D**,**E**) represent the mean of CT value in unstented area and all stents with these scanning protocols.

**Figure 2:**
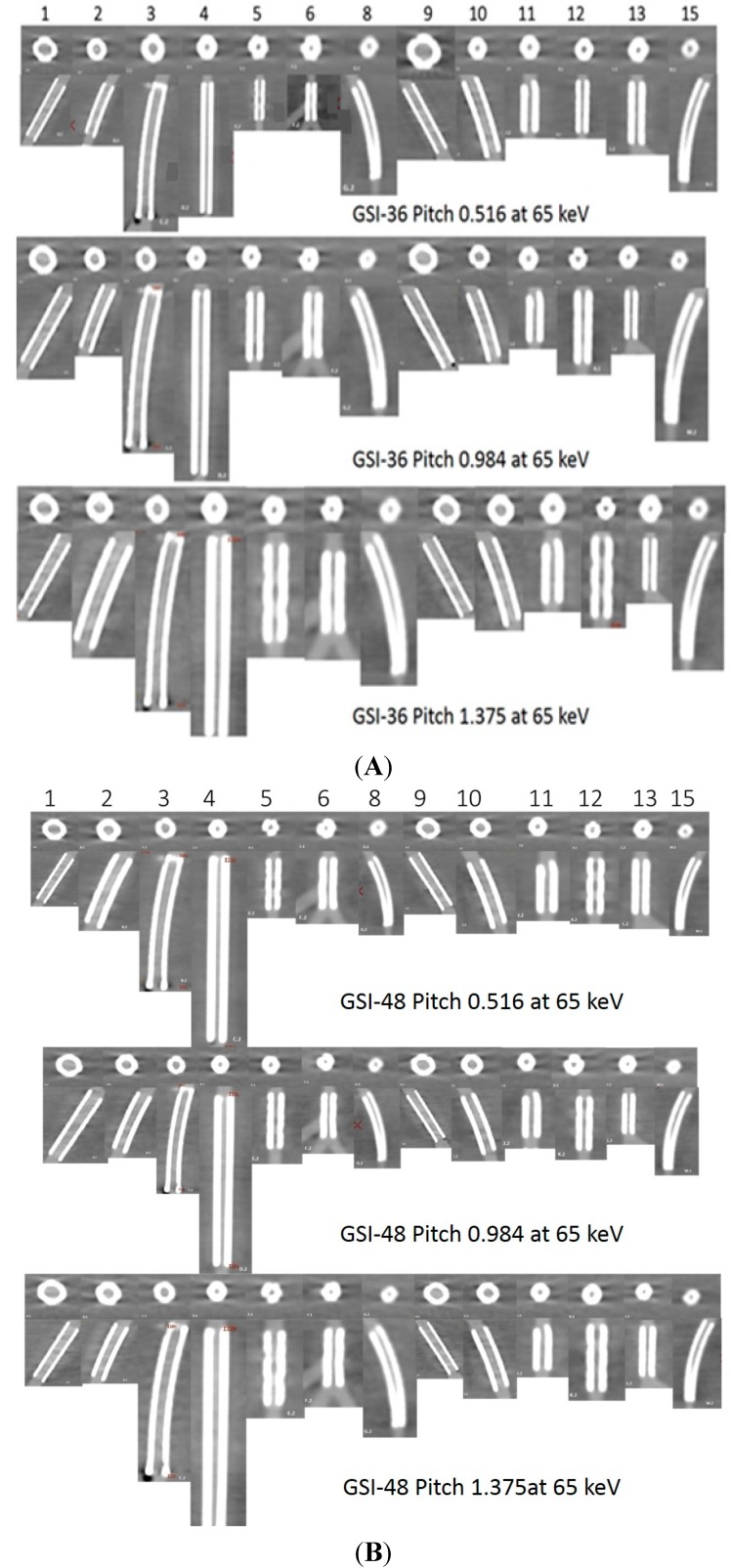

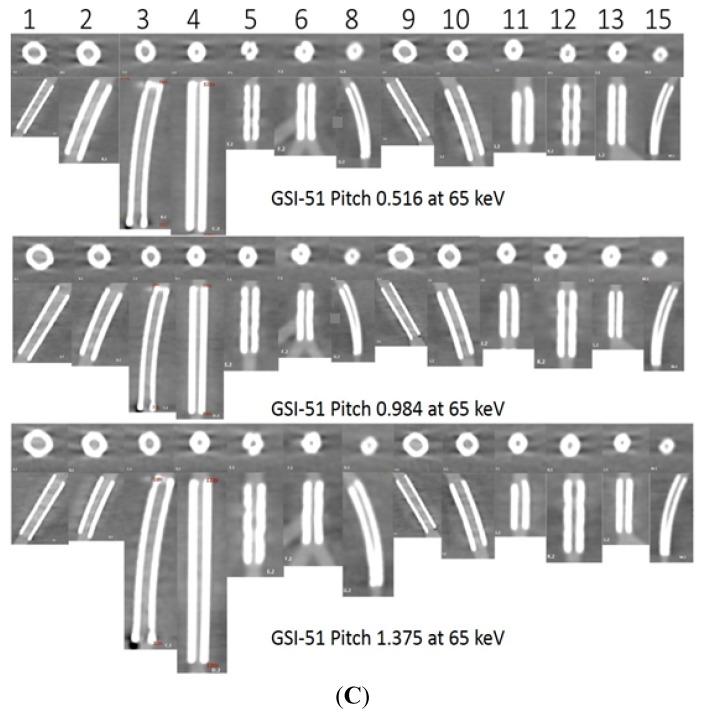
A total of 13 stents (No. 7 and 14 were not included due to difficulty placing region of interest in the area) with axial and coronal reformatted images were demonstrated with three GSI protocols (**A**–**C**: GSI-36, GSI-48, and GSI-51, respectively) and three pitch values at a keV of 65.

A total of 180 series acquired with virtual MEI imaging at 4-kiloelectron volts (keV) and 5-adaptive statistical iterative reconstruction (ASIR) levels were reconstructed to determine the interaction between peripheral arterial stent image quality factors and scanning protocols. There was a significant reduction of image noise with MEI between 65 and 70 keV in all investigated preset GSI protocols (*p* < 0.05). A significant reduction was observed at 65 keV for the unstented area, and for large diameter stents and small diameter stents with both GSI-48 and GSI-51. However, the mean HU was reduced as the keV increased for all protocols, as shown in Figure 1E. Results indicated that the preset GSI-48 scanning protocol with a pitch value of 0.984, 65 keV, and ASIR ≤ 50% achieved the optimal image quality compared with the other protocols, as shown in Figure 1A–E. Figure 2 shows an example of a series of images acquired with protocols using 65 keV and 3 GSI settings. 

### 2.1. Image Quality Assessment

**Figure 3:**
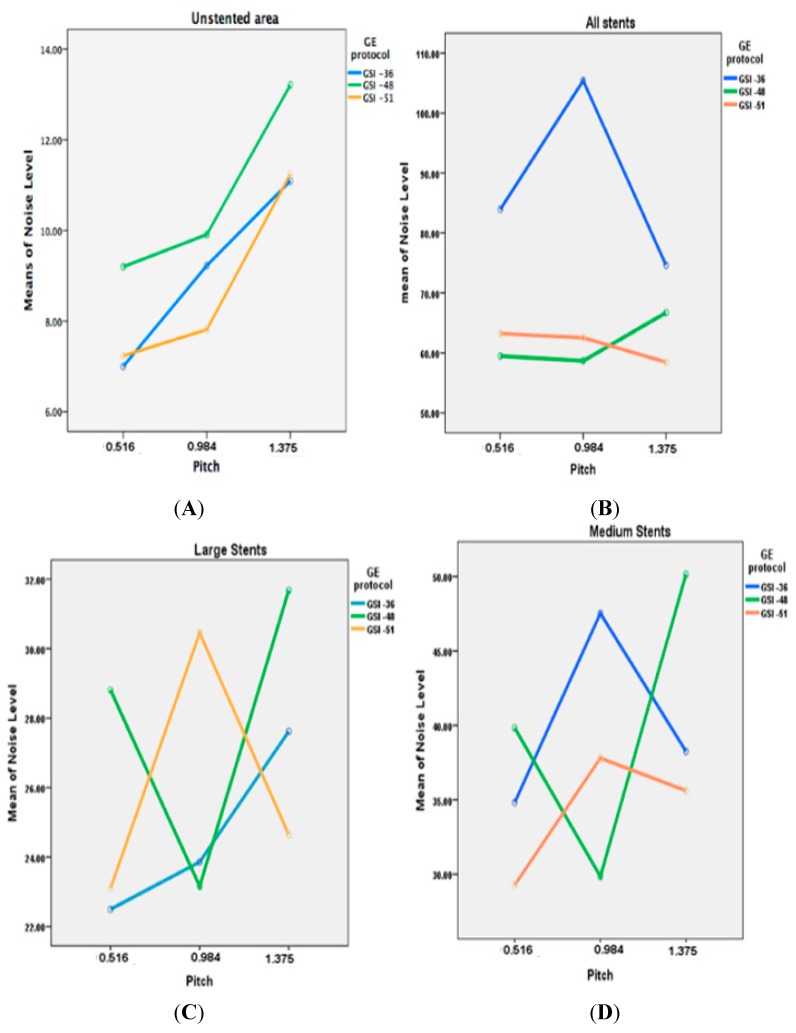

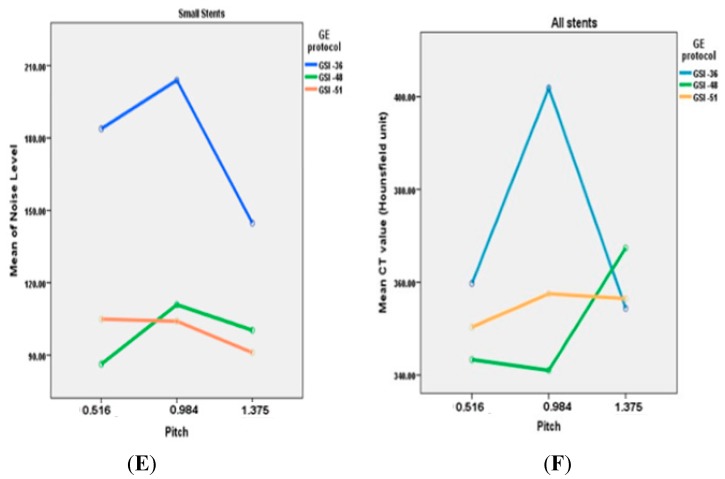
(**A**–**E**) A comparison of relationship between noise levels measured with different GSI protocols and pitch values at 65 keV with different diameters of stents; (**F**) represents the mean of CT values measured in all stents with use of three GSI and pitch protocols.

The phantom data were analyzed with a 3-GSI * 3-Pitch * 4-keV factorial ANOVA. Each effect was tested with a mean standard error (MSE) of 71.27. There was highly significant interaction of image noise and SNR with the GSI and pitch (*p* = 0.001). Similar findings were observed in the unstented area with highly significant effects. In addition, significant effects were found for these factors—GSI, pitch, and keV (*p* = 0.001). In contrast, there was another significant interaction on the unstented area between GSI and ASIR (*p* = 0.015) and a very highly significant difference between keV and ASIR (*p* < 0.001). Although for noise level, two of the 3-way interactive terms were statistically significant, namely the GSI, pitch, and keV, and the GSI, pitch, and ASIR (*p* < 0.001); the main effects and interactions for these are shown in Table 1. The noise level (MSE = 89.04) was higher in GSI-36 than in the other two GSIs, as shown in Figure 3A–E.

**Table 1:** Results of factorial ANOVA.

Effects		SNR		SNR2		NL		NL2	
		*F*	*P*	*F*	*P*	*F*	*P*	*F*	*P*
GSI	Main effect	11.806	0.001	86.658	0.000	947.509	0.000	572.661	0.000
Pitch	Main effect	10.992	0.002	302.411	0.000	102.664	0.000	1883.182	0.000
KeV	Main effect	13.424	0.000	192.161	0.000	230.042	0.000	894.644	0.000
ASIR	Main effect	NA	NA	157.416	0.000	NA	NA	1291.264	0.000
GSI * ASIR	Two-factor interaction effect	NA	NA	3.823	0.015	NA	NA	27.164	0.000
KeV * ASIR	Two-factor interaction effect	NA	NA	6.133	0.001	NA	NA	3.652	0.010
Pitch * ASIR	Two-factor interaction effect	NA	NA	1.877	0.147	NA	NA	68.25	0.000
GSI * KeV	Two-factor interaction effect	0.454	0.829	16.528	0.000	0.649	0.691	120.311	0.000
GSI * Pitch	Two-factor interaction effect	13.708	0.000	12.793	0.000	142.367	0.000	30.586	0.000
Pitch * KeV	Two-factor interaction effect	0.419	0.853	18.778	0.000	1.189	0.375	120.391	0.000
GSI * KeV * ASIR	Three-factor interaction effect	NA	NA	0.457	0.920	NA	NA	1.727	0.123
GSI * Pitch * ASIR	Three-factor interaction effect	NA	NA	0.634	0.742	NA	NA	9.842	0.000
Pitch * KeV * ASIR	Three-factor interaction effect	NA	NA	0.558	0.853	NA	NA	1.326	0.268
GSI * Pitch * KeV	Three-factor interaction effect	NA	NA	21.545	0.000	NA	NA	118.371	0.000

SNR: Signal to noise ratio in the stented area; SNR2: Signal to noise ratio in the unstented area; NL: noise level in the stented area; NL2: noise level in the unstented area; *F*: value of test statistic of *F*-test for corresponding effect; *P*: corresponding *p* value; GSI: gemstone spectral image protocol; keV: kiloelectron volt; ASIR: adaptive statistical iterative reconstruction; NA: not applicable; * = multiplication.

### 2.2. Effect of keV on Image Quality

**Figure 4:**
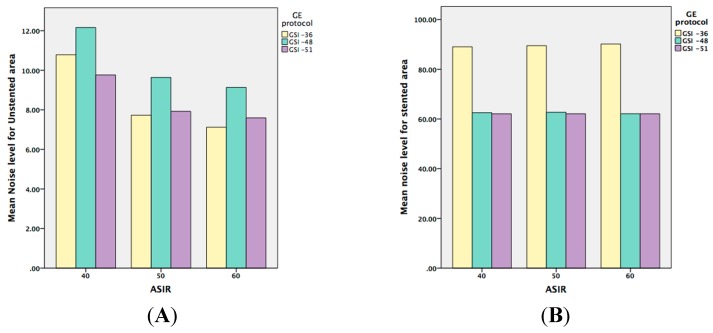

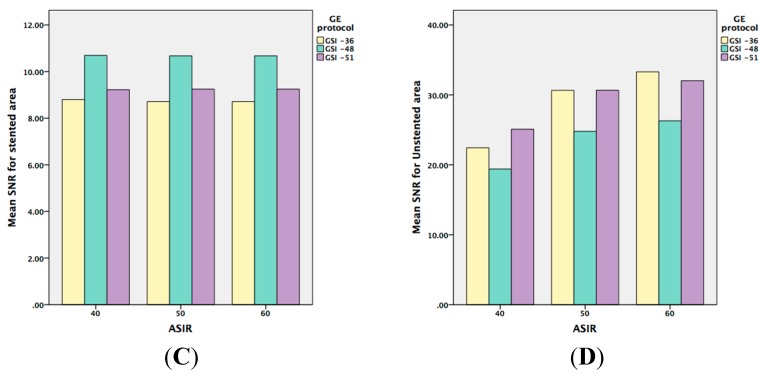
(**A**,**B**) are graphic representations showing the noise level when ASIR is used within the both unstented and stented areas; while (**C**,**D**) represent the means of SNR when ASIR is used.

There was no significant interaction between keV and GSI or pitch factors in the stented area. However, this does not mean that there was no effect of keV on image quality, but the effect of keV on image noise was not dependent on GSI or pitch. Therefore, the four noise-level means for keV ranged from 83.94 to 64.30 HU and, incidentally, decreasing image noise occurred monotonically with increasing keV. Thus, the effect of keV on image noise was independent of any other design effect. A similar effect was observed with the SNR. Unlike its effect on the unstented area, the effect of keV was found to be highly significant for both image noise and SNR (*p* = 0.001). Figure 4 compares the selected keV in different GSIs for both unstented and stented areas with different ASIR values.

### 2.3. Effect of GSI and Pitch on Image Quality

The interaction of GSI by pitch is highly statistically significant (*p* < 0.001). A significance interaction was observed on the unstented area. Also, the main effects of GSI and pitch on noise level are highly significant (*p* < 0.001), as shown in Table 1. 

The marginal noise level means for the 3-GSI categories were 89.03, 62.86, and 61.92, indicating significant differences with the first GSI and with both the second and third GSI of around 27.11 and 0.94 HU, respectively. However, when the three GSI means were investigated for the first pitch category (84.90, 60.60, and 63.95), significant changes were observed. These changes varied both with respect to magnitude and direction, and presented as the significant interaction of GSI by pitch.

### 2.4. Effect of Type of Stents on Image Quality

The noise level in the unstented area showed a direct relationship between image noise and pitch value, with noise increasing when pitch increased. However, with large stents, lower image noise was found in GSI-36 and GSI-48 protocols, with a pitch value of 0.984. The medium sized stent showed that a lower noise was achieved by a GSI-48 and pitch of 0.984, while result is different from that for the small stents where the best visualization was achieved with use of GSI-51 and pitch of 0.516, as shown in Figure 3. 

A radiation dose reduction of about 50% was achieved in all protocols when the pitch value 0.984 was used. However, a minimal reduction was observed when the pitch value was changed from 0.984 to 1.375.

### 2.5. Subjective Image Quality Assessment

**Figure 5:**
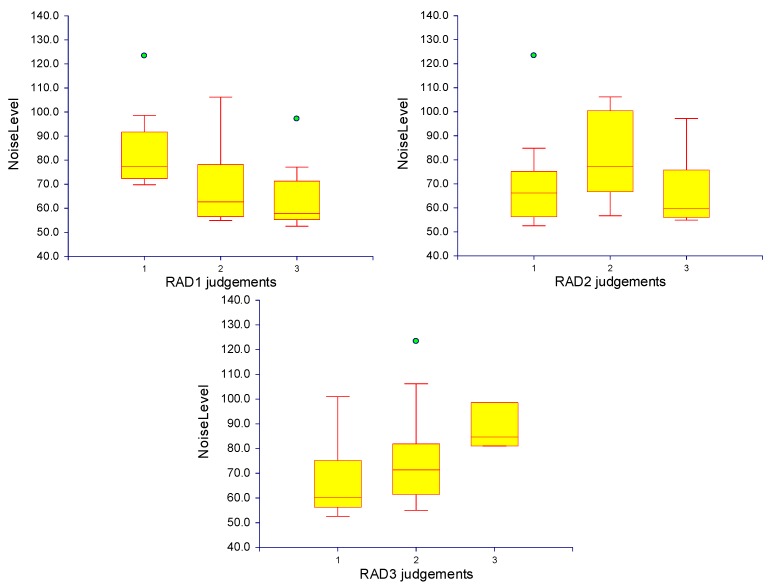
Box plots demonstrate the radiologist’s evaluation of the image quality using a 3-point scale. The red line within the yellow box indicates the median of the values. The red line at the bottom of the box corresponds to the 25th percentile, and the one at the top is the 75th percentile. The range between these two lines (*i.e.*, the length of the box) is the inter Quartile Range (IQR). The lower cross line is 1.5 IQR below the 25th percentile, the upper cross line is 1.5 IQR above the 75th percentile. Individual observations beyond either of these cross line are outliners, shown as green circles.

The subjective grading of image quality showed a discrepancy between the readers with a kappa value of 0.24. This might be explained by the limited experience of those readers in clinical research. The box plots of the noise level for the three radiologists are shown in Figure 5.

## 3. Discussion

This phantom study indicates that the effect of the pitch factor on the keV and radiation dose is an important indicator for determining both radiation dose and image quality, as the lowest pitch is associated with the highest radiation dose and *vice versa*. The lowest radiation dose with sufficient image noise was obtained with the GSI-48 protocol with a pitch of 0.984 and 65 keV. Overall, the GSI-51 protocol achieved the highest SNR and lowest noise level values with pitch values of 0.516 and 1.375; however, a pitch value of 0.516 was associated with the highest radiation dose, whereas a pitch value of 1.375 was associated with the highest image noise. To our knowledge, no studies have been published comparing different DECT protocols with different pitch values. Results of this study show that the effect of the pitch value on image noise was strongly dependent on rotation time, as the protocol with the shorter rotation time (GSI-36) achieved both the highest SNR and the lowest image noise, and the protocol with the longer rotation time (GSI-51) achieved the highest image noise, even when mAs was higher than that of the other protocols. 

It was found that the images acquired with approximately 65–70 keV had less image noise and higher SNR compared to other energies that have a lower noise level and lower SNR. Our results are similar to those reported from a chest study by Cheng *et al* [15] who found that the MEI images at 65–70 keV resulted in less image noise and a better contrast-to-noise ratio. The findings of the current study are consistent with those of Yu *et al* [21], who studied various phantom sizes to evaluate MEI at multiple keV levels to optimize chest image quality. They found that the best image quality was obtained with energies of 66 keV for small phantoms, 68 keV for medium, 70 keV for large, and 72 keV for extra-large phantoms [21]. Similarly, Matsumoto *et al.* [22] reported that using 70 keV achieved the lowest image noise based on a phantom study. Furthermore, Pehno *et al* [13] compared the subjective and objective image quality of virtual MEI DECT angiography (DECTA) to conventional polyenergetic images (PEI) in aortoillic arteries, demonstrating optimal contrast enhancement and improved image quality using 70 keV MEI compared to single-energy CTA. However, our findings show that the highest image noise was found with 60 keV, which differs from those reported by Sudarski *et al.* [23]. They found that the use of 60 keV for lower extremities led to the best image quality when compared to the quality of PEIs. These findings can be justified because the effect of stents on image noise is clearly evident when comparing small, medium, and large stents with different keV, as shown in Figure 5. The findings from these studies suggest that using keV between 65 and 70 with a pitch value of 0.984 achieves optimal image quality with a lower radiation dose in peripheral arterial DECT.

When iterative reconstruction is evaluated there is a significant difference between the unstented and stented areas. In the unstented area, the image quality was improved when the ASIR was increased from 40% to 50%. This is similar to previous studies that showed that an ASIR of less than 40% did not improve the image quality when compared with conventional PEIs [14,24,25,26]. However, the current study shows that images with stents were not affected by any level of ASIR when they were applied with all of the preset GSI protocols. Therefore, based on the unstented area results, we recommend the use of 50% ASIR, with 65 to 70 keV in the peripheral arterial stent protocol, and preset GSI-48 as the optimal protocol to replace the conventional CTA.

The optimal protocol that has been evaluated and identified in this study is currently being tested in a clinical study to validate its clinical value and outcome. Despite these protocols being tested on a GE scanner, results of this study can be applicable to other MDCT manufacturers as long as the dual-energy function is available on these scanners.

Our study has some limitations. First, although the experimental setup was developed to simulate a peripheral vascular tree, the idealized anatomic environment did not have surrounding organs, vessel walls, or tissues. The nature of body vessels varies from those in the phantom, and this could affect visualization of stents to some extent. Another limitation is that this custom-made phantom represented only an average-sized adult, while absorption of low-energy radiation will differ for large- or small-sized patients. Finally, the default manufacturer’s setting of 0.3 as the weighting factor for the low-energy tube was used to create virtual 120 KV reconstructions; however, there have been reports that a weighting factor of 0.5 improves image quality and therefore would be better for vascular imaging [27]. This suggests that further studies are necessary to confirm our findings. 

## 4. Experimental Section

### 4.1. Peripheral Artery Phantom Design and Stent Placement

**Figure 6:**
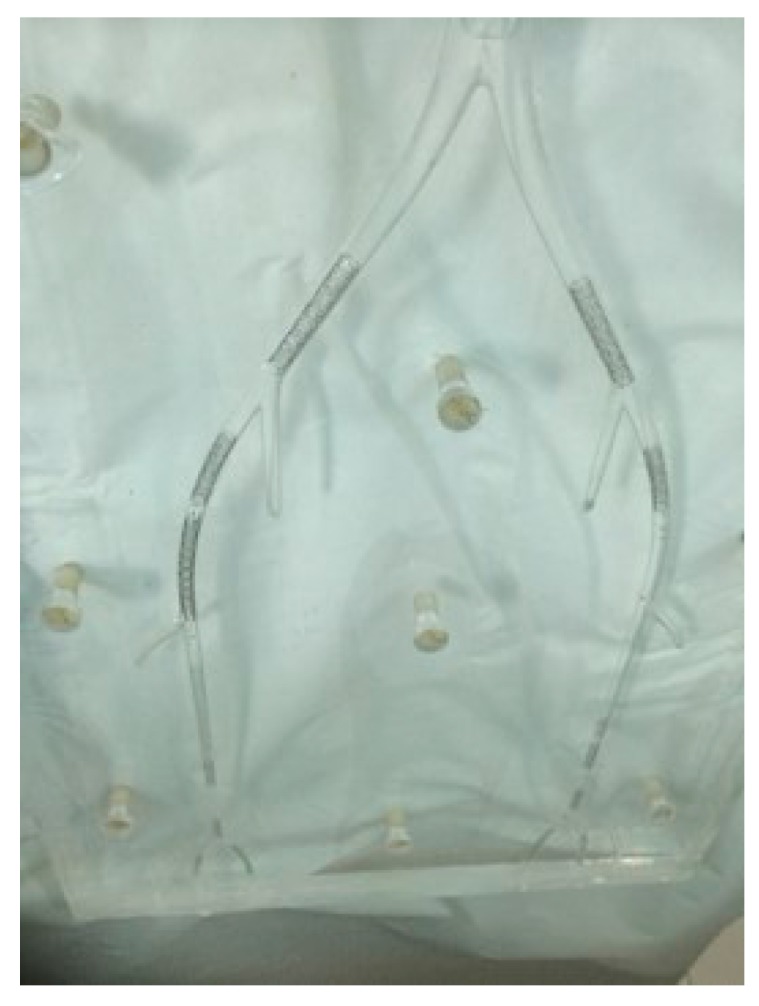
Photograph of the phantom with stents insertion.

The custom-made peripheral arterial phantom consisting of a main peripheral arterial tree and arterial branches was developed with use of a computer-aided design program to represent realistic anatomic dimensions. The phantom was made of poly methyl methacrylate material with anatomical dimensions similar to the normal anatomy of a peripheral arterial tree, as shown in Figure 6.

A total of 15 expired stents with different sizes (diameters), materials, and designs were used in the experiments. Details of the stents are shown in Table 2. Of these fifteen stents, 10 were made of stainless steel (316 L), 3 of a platinum chromium alloy, 1 of Nitinol, and 1 of cobalt-superalloy. Stents were deployed into the simulated peripheral arteries modeled within the phantom. Stents with lumen diameters ranging from 2.75 to 3, 4, 5, 6, 7, and 8 mm, which closely matched their nominal diameter, were inserted into the arteries with two exceptions: the two Taxus Element stents with nominal diameters of 3 mm were inserted into simulated arteries with a diameter of 2.5 mm.

### 4.2. DECT Scanning Protocols and Image Reconstruction

DECT scans were performed with a fast kilovoltage-switching 64-slice CT scanner (Discovery CT750 HD; GE Healthcare, Milwaukee, WI, USA). Of the GSI-protocols that the manufacturer has developed as default settings, 3 GSI protocols (GSI preset protocols 36, 48, and 51) were selected for this study based on the lowest CTDI_vol_ with acceptable diagnostic image quality to evaluate peripheral arterial stents. These GSI preset protocols have been fixed by the manufacturer to maintain constant tube power when switching the tube voltage back and forth. Therefore, changing the radiation dose in these preset protocols was only possible by adjusting the pitch and/or rotation time. As a result, the three available pitch values, 0.516, 0.984, and 1.375 were tested, with a beam collimation of 40 mm in all of these protocols and fixed rotation times of 0.5, 0.7, and 0.8 second per rotation, respectively, for each protocol. The phantom was then positioned in the gantry in an orientation parallel to the *z*-axis of the scanner.

The scanning parameters for the selected preset GIS-protocols are summarized in Table 3. Images were acquired with coverage of 30 cm ranging from aortic bifurcation to arteries below knee. The raw data obtained from each scan were reconstructed in four image sets with five levels of adaptive statistical iterative reconstruction (ASIR), 0%, 30%, 40%, 50%, and 60%, and four keV of 60, 65, 70, and 75, respectively, with slice thicknesses of 1 mm with 50% reconstruction overlap. Tube current modulation was not available in dual energy acquisition in this system for all protocols.

**Table 2:** Details of the examined stents.

Model	Material	Manufacturer	Diameter (mm)	Length (mm)	Stent Diameter	Stents No.
Express LD	316 L stainless steel	Boston Scientific	7–8	27–37	Large	(1, 2, 9 and 10)
Absolute .035	Nitinol	Abbott	6	40	Medium	3
Wallstent-Uni Endoprosthesis	Cobalt-superalloy	Boston Scientific	5	40	Medium	4
Palmaz Genesis	316 L Stainless steel	Cordis	5	14	Medium	(5, 6 and 11)
Taxus Element	316 L Stainless steel	Boston Scientific	2.75	32	Small	(7, 14)
Taxus Libert 2nd Generation	Platinum Chromium	Boston Scientific	3	28	Small	8
Promus Element	Platinum Chromium	Boston Scientific	4	16	Medium	12
Express Vascular SD	316 L Stainless steel	Boston Scientific	4	15	Medium	13
Monorail Liberté	316 L Stainless steel	Boston Scientific	3	28	Small	15

**Table 3:** Details of scan parameters by protocols.

Scan Parameters	Protocol 1	Protocol 2	Protocol 3
GSI protocol	GSI-36	GSI-48	GSI-51
Scan mode	Dual-energy	Dual-energy	Dual-energy
Tube potential	80/140 kV	80/140 kV	80/140 kV
Tube current	260 mAs	260 mAs	360 mAs
Rotation time (s)	0.8	0.7	0.5
Detector collimation (mm)	64 × 0.625	64 × 0.625	64 × 0.625
Pitch	0.516, 0.984 and 1.375	0.516, 0.984 and 1.375	0.516, 0.984 and 1.375
Table speed (mm/R)	20.62	39.37	55
Reconstruction kernel		Standard	
Section thickness (mm)	1	1	1
Interval	0.5	0.5	0.5
keV		(60, 65, 70, and 75)	
ASIR		(30, 40, 50, and 60)	

A simulated intravenous contrast medium (Omnipaque 350, GE Healthcare, Milwaukee, WI, USA) was used to represent the actual contrast-enhanced CT angiography. The contrast medium was diluted with normal saline to reach the attenuation of 250 Hounsfield unit (HU), which is the acceptable CT attenuation in peripheral CT angiography. The contrast medium was injected into the simulated arteries, which were sealed at both ends [28].

### 4.3. Quantitative Image Assessment

**Figure 7:**
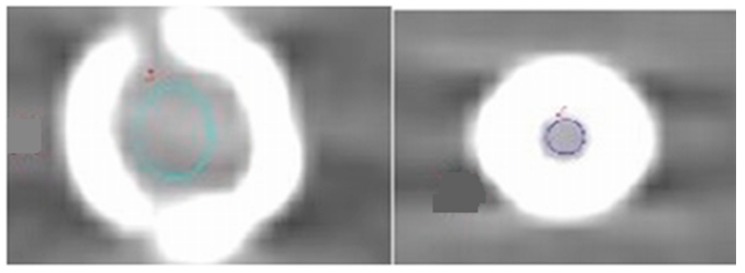
The region of interest was placed in the inner stent lumen for measurement of image noise. The colored circles indicate measurement inside the stent lumen.

Quantitative measurements were performed for DECT images at 1 mm slice thickness on a separate independent workstation with the GSI Viewer (ADW 4.6 General Electric Healthcare, Milwaukee, WI, USA). The mean HU was obtained by placing a circular region of interest (ROI) in selected areas of the phantom for the 15 stents (iliac arteries, common femoral arteries, superficial femoral arteries, popliteal arteries, anterior tibial arteries, peroneal arteries, and posterior tibial arteries). Two ROIs were selected; the first one was placed in the common femoral artery to measure the noise on an unstented area of the phantom. The second ROI was placed in the stented lumen area on the axial images to measure the noise for all stents without inclusion of stent struts, as shown in Figure 7. For two stents—No. 7 and No. 14—it was difficult to place the ROI due to the small size of the stents with a limited lumen area being visualized; therefore, we excluded them from the analysis.

The selected ROI in an unstented area was in the femoral artery and within each stent at the same location for all protocols to measure the mean CT attenuation and also image noise that was defined as the standard deviation (SD). Moreover, the signal-to-noise ratio (SNR) for both the stented and unstented areas was calculated with the following formula:

SNR = in stent lumen or nonstented area of CT attenuation in HU/SD
(1)

### 4.4. Qualitative Assessment of Image Quality

Three radiologists with 5, 15, and 20 years of experience in CT imaging, respectively, performed qualitative evaluation on the GSI viewer with identical window width, window level, and FOV. Different MEI series from the each GSI protocol were evaluated randomly (between 60, 65, 70 and 75 keV within each ASIR), with a total of 180 series reviewed by each reviewer. The readers were blinded to all scanning parameters, except for the different energies, because different values of keV could easily be determined by visual inspection of the images. Intraobserver variability was not estimated because each radiologist assessed the images only once. Visualization of the stent lumen was assessed using a 3-point scale (1 = poor image quality and non-diagnostic; 2 = adequate image quality; and 3 = good image quality).

### 4.5. CT Dose

A fixed scan length of 29.9 cm was used for all examinations. The volume CT dose index (CTDIvol) and dose-length products (DLP) were recorded for calculation of effective dose, as shown in Table 4. Effective dose (ED) was calculated for each protocol using a conversion factor of 0.015, which is taken from the normalized value of the effective dose per dose-length product for peripheral arteries [29].

**Table 4:** Summary of CTDI_vol_ values, dose-length products, and effective doses across the protocols.

GE Protocol	Pitch	CTDI_vol_ (mGy)	DLP (mGy × cm)	Effective Dose (mSv)
GSI-36	0.516	39.33	863.86	12.96
GSI-36	0.984	10.30	457.56	6.86
GSI-36	1.375	7.73	341.00	6.47
GSI-48	0.516	17.28	759.13	11.39
GSI-48	0.984	9.05	402.10	6.03
GSI-48	1.375	6.48	299.72	4.50
GSI-51	0.516	19.74	867.57	13.01
GSI-51	0.984	10.34	459.66	6.89
GSI-51	1.375	7.40	342.69	5.14

GSI, gemstone spectral imaging; CTDI_vol_, computed tomography volume dose index; DLP, dose-length product.

### 4.6. Statistical Analysis

All of the data were entered into SPSS Statistics version 22.0 (SPSS Inc., Chicago, IL, USA) for statistical analysis. Two data sets—the noise level and SNR (independent variable) responses obtained when ASIR = 0—were used to produce 36 observations in a 3-factor factorial analysis of variance (univariate ANOVA) model: GSI (3-levels: GSI-36, GSI-48, GSI-51), pitch (3-levels: 0.516, 0.984, 1.375), and keV (4-level: 60, 65, 70, 75).

The second part of the statistical analysis relates to the noise level and SNR in the nonstented area when ASIR ≥ 40; 3-levels: 40, 50, 60 were used to produce 108 observations in a 4-factor factorial model. The highest order interaction in each analysis was employed as an estimate of residual (error) variation; therefore, when there was no significant interaction, the main effect was considered. Statistical significance was assessed by comparing the *p* values obtained in the *F*-tests corresponding to the main effect of GSI as well as to the interaction effect of pitch, keV, and ASIR. All tests were performed at the 5% significance level.

## 5. Conclusions

In conclusion, all preset GSI protocols were found to be suitable for the evaluation of peripheral arterial stents. This study recommends use of the faster rotation time with a pitch value of 0.984 and keV of 65–70 with 50% ASIR for peripheral arterial stent visualization with DECT, as this protocol results in both lower image noise and radiation dose, but with acceptable diagnostic images.
